# Association between *TGFB1* genetic polymorphisms and chronic allograft dysfunction: a systematic review and meta-analysis

**DOI:** 10.18632/oncotarget.19516

**Published:** 2017-07-24

**Authors:** Kun Liu, Xuzhong Liu, Shuo Gu, Qing Sun, Yunyan Wang, Junsong Meng, Zongyuan Xu

**Affiliations:** ^1^ Department of Urology, Huai’an First People’s Hospital, Nanjing Medical University, Huai’an, 223300 China

**Keywords:** TGFB1, polymorphism, chronic allograft dysfunction, meta-analysis

## Abstract

**Background:**

Epidemiological studies have investigated the role of transforming growth factor-β1 (TGF-β1) in chronic allograft dysfunction (CAD) following kidney transplantation. *TGFB1* gene polymorphisms (SNP rs1800470 and rs1800471) may be associated with the risk of CAD. In this meta-analysis, the relationship between these two variations and the risk of CAD was explored.

**Materials and Methods:**

MEDLINE, EMBASE, the Cochrane Central Register of Controlled Trials (CENTRAL), Embase, the Chinese CNKI and WANFANG databases were searched. Data were extracted and pooled results were estimated from odds ratios (ORs) with 95% confidential intervals (95% CIs). Quality assessments were performed, and publication bias of all eligible studies examined.

**Results:**

Eight studies with 1038 subjects were included in our analysis. According to the effects on TGF-β1 secretion, haplotypes were categorized as “HIGH”, “INTERMEDIATE” and “LOW”. The combined results showed a statistically significant difference of *TGFB1* haplotypes between the CAD recipients and control subjects when “HIGH” with “INTERMEDIATE” and “LOW” (“HIGH” vs. “INTERMEDIATE” + “LOW”: OR: 3.56, 95% CIs: 2.20, 5.78, P < 0.001) were compared. No significant association was found between the *TGFB1* codon 10 or codon 25 and the CAD risk in all five genetic models.

**Conclusions:**

Our meta-analysis has found the haplotype of *TGFB1* codon 10/25 T/T G/G and T/C G/G genotypes, associated with increased production of TGF-β1, was linked with CAD risk following kidney transplantation. Moreover, no significant difference was found between TGFB1 codon 10 or codon 25 and the development of CAD.

## INTRODUCTION

Kidney transplantation is the optimal therapy for end-stage renal disease [1]. Short-term allograft survival has been significantly improved due to advancements in immuosuppressive agents and surgical techniques [2]. However, long-term allograft survival, especially in the period of 10 to 20 years after kidney transplantation, has not accommodated the increase of short-term survival. Numerous studies have shown that chronic allograft dysfunction (CAD) is the main cause of graft failure [3, 4].

CAD is mainly characterized as glomerular sclerosis, tubular atrophy and interstitial fibrosis [5]. Various factors contribute to the pathogenesis of CAD, such as chronic calcineurin inhibitor (CNI), particularly cyclosporine A (CsA) nephrotoxicity, fibrosis-related cytokines and immune-associated factors [6–8]. Among these factors, certain cytokines, including transforming growth factor-β1 (TGF-β1), tumor necrosis factor-α (TNF-α) and cell tissue growth factors (CTGF), were considered to be closely associated with the development of fibrogenesis, thus contributing to the pathogenesis of CAD [5, 9, 10]. Recent studies have reported that TGF-β1 could promote the development of interstitial fibrosis and tubular atrophy through epithelial-to-mesenchymal transition (EMT), endothelial-to-mesenchymal transition (EndMT) and other mechanisms [5, 11].

TGF-β1 is a multifunctional growth cytokine in humans and one of the significant mediators of wound healing and tissue regeneration [12]. *TGFB1* is mapped on to chromosome 19q13.1–13.3 with seven exons and six introns, and the expression of *TGFB1* and production of TGF-β1 is related to single nucleotide polymorphisms (SNPs). *TGFB1* contains two SNPs, +869T/C at codon 10 (rs1800470) and +915G/C at codon 25 (rs1800471) contributing to variation in TGF-β1 production both *in vitro* and in serum [13]. There is increasing evidence that the level of production of a series of cytokines can be modulated by polymorphisms in the corresponding genes [14]. Since fibrosis-related cytokines play an important role in the inflammatory and immune response that mediate the outcome of kidney transplantation, the relationship between those cytokine polymorphisms, particularly *TGFB1* SNPs, and CAD has been explored in several studies [15–17]. However, the results of these studies were inconsistent and often conflicting.

Based on the crucial role of TGF-β1 in CAD pathogenesis, we performed meta-analysis to investigate the contributions of two TGFB1 SNPs to CAD risk.

## MATERIALS AND METHODS

### Search strategy

A comprehensive literature search was performed in PubMed, the Cochrane Central Register of Controlled Trials (CENTRAL), Embase, the Chinese CNKI and WANFANG databases (updated on December 20st 2016) by two independent authors (Kun L and Xuzhong L). The following keywords were used: (transforming growth factor-beta 1 OR TGFB1), AND (polymorphisms OR SNPs OR variants), AND (chronic rejection OR chronic allograft nephropathy OR chronic allograft dysfunction), AND (MESH item, kidney transplantation). The equivalent Chinese terms were used in the Chinese databases. Furthermore, the reference lists of all studies included in the meta-analysis were also reviewed for possible inclusion.

### Inclusion and exclusion criteria

Studies were included if they met the following eligibility criteria: (1) case-control studies designed to investigate the relationship between *TGFB1* SNPs and chronic allograft dysfunction after kidney transplantation; (2) available information on the frequencies of genotype or allele in case and control groups; (3) all subjects from three allelic groups were derived from a population within the same geographic area and ethnic background; (4) full-text article were published in English or Chinese. The exclusion criteria were: (1) Case reports; (2) reviews; (3) animal experiment, chemistry, or cell line studies; (4) studies in a language other than English or Chinese; (5). No eligible or insufficient data frequencies of genotypes for each polymorphism could be extracted for meta-analysis. Two authors (Kun L and Xuzhong L) independently assessed and selected trials for final analysis with discrepancies resolved by consensus.

### Data extraction

Two investigators (Kun L and Xuzhong L) independently extracted relevant data from all selected studies and reached consensus for all items. Basic characteristics of patients were collected as following: first author’s name, year of publication, nation/race, number of subjects, the proportion of male subjects, mean age and method of genotype. In addition, the genotype and allele frequencies of *TGFB1* SNPs were collected using a standardized data extraction form. Missing data was examined by contacting the first or corresponding author.

### Quality assessment

The methodological quality of each included study was assessed by the Newcastle-Ottawa quality assessment scale (NOS)(18). Each study was evaluated on the standard criteria and categorized based on three factors, including selection, comparability and exposure. Scores ranged from 0 to 9; 9 points represented the highest quality and lowest risk of bias.

### Statistical analysis

The pooled data was used to assess the strength of the association between *TGFB1* polymorphisms and CAD using odds ratio (OR) with 95% confidence intervals (95% CIs) in a dominant model, a recessive model, a co-dominant model, a co-recessive model and an allele model. A *p value* less than 0.05 was considered statistically significant. Heterogeneity test among trials was determined by *I*^2^, and was defined as 100% *(Q-df )/Q, where Q is Cochran’s heterogeneity statistic and df is the degrees of freedom, with a fixed-effect model set at low statistical inconsistency (*I*^2^ < 25%). Otherwise, a random-effects model was selected, which is better adapted to clinical and statistical variations [18–19]. To explore the potential effects of heterogeneity, stratification analysis by ethnicity, age and quality criteria was carried out. The Egger’s regression test and funnel plots were used to assess potential publication bias. Cumulative meta-analysis was carried out by the year of publication. All analyses were performed using STATA (release 12.0, College Station, TX).

## RESULTS

### Literature selection and study characteristics

**Figure 1:**
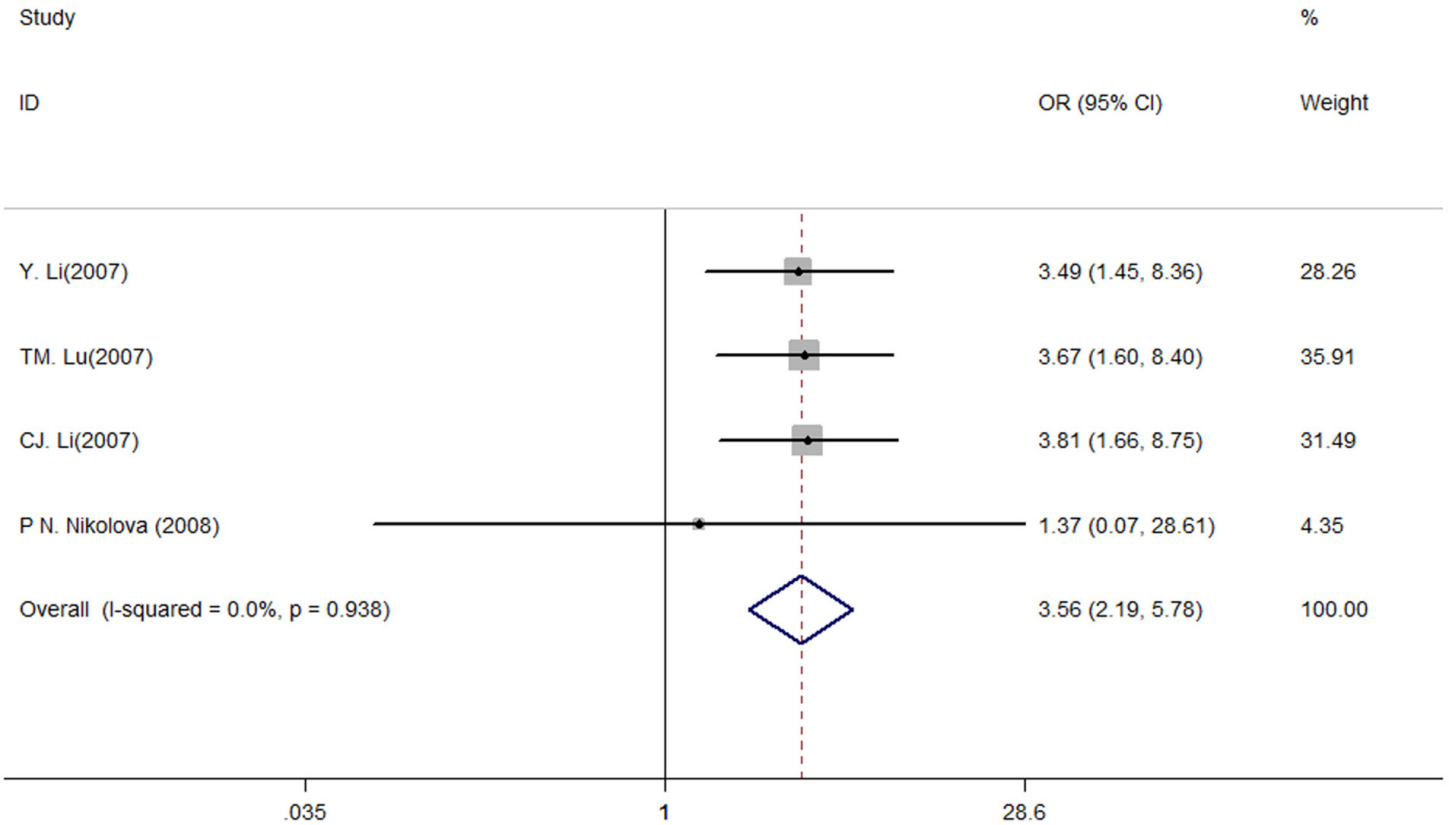
Flow chart of literature search and study selection

A flow diagram of the screening process for included studies is shown in Figure 1. Primary screening identified 18 potentially relevant articles, including 15 articles in English and 3 in Chinese. Based on their titles and abstracts, 8 articles were excluded due to unrelated subject or no available data. After screening the full articles, a total of 8 trials with 1038 subjects met the criteria and were included in our meta-analysis.

Basic characteristics of eligible studies are shown in Table 1. Overall, the meta-analysis of included studies comprised of *TGFB1* codon 10 and codon 25. Four studies [17, 20–22] included patients with CAN, confirmed by renal biopsy. CAD recipients included in another 4 trials were evaluated by the clinical examinations. The 8 studies included 4 Asian [20–23], 3 European [15, 17, 24] and 1 Latino [25] populations. Results of quality assessment using the NOS scale is presented in Table 2.

**Table 1:** Characteristics of included studies in the meta-analysis

Author (year)	Ethnicity	Case number	Age (years; mean ± SD)	Male/female	Genotyping method	Genetic equilibrium	Diagnosis criteria
**A. Melk (2003)**	Caucasian	105	Con:8.6 ± 4.4; Case:9.2 ± 4.3	57/48	RT-PCR	NA	CAD
**J Y. Park(2004)**	Asian	163	NA	NA	PCR-SSCP	NA	CAN
**K. Gendzekhadze(2006)**	Latino	62	40 ± 10	37/26	PCR-SSP	Yes	CAD
**Y. Li(2007)**	Asian	122	39.7	95/27	PCR	NA	CAD
**TM. Lu(2007)**	Asian	144	40.6	109/35	PCR-SSP	NA	CAN
**CJ. Li(2007)**	Asian	100	Con: 42.8 ± 7.9;Case:45.3 ± 6.7	51/49	PCR	NA	CAN
**P N. Nikolova (2008)**	Caucasian	66	NA	39/27	PCR-SSP	NA	CAN
**M A. Jiménez-Sousa (2012)**	Caucasian	276	Con:50.5 ± 15.2;Case:51.5 ± 18	108/168	the SNPlex genotyping system	Yes	CAD

Abbreviations: SD, standard deviation; NA, not available; CAD, chronic allograft dysfunction; CAN, chronic allograft nephropathy; PCR-SSCP, PCR-single strand conformation polymorphism; PCR-SSP, PCR-sequence-specific primers.

**Table 2:** Newcastle-Ottawa quality assessment scale for each included study

Studies	Selection				Comparability		Exposure			Total quality score
	**Case definition adequate**	**Representativeness of the cases**	**Selection of controls**	**Definition of controls**	**Adjustment for age**	**Adjustment for lifestyle/traditional risk factors**	**Ascertainment of exposure**	**Uniform method of ascertainment**	**Non-response rate**	
**A. Melk (2003)**	1	1	1	1	1	1	0	1	0	7
**J Y. Park(2004)**	1	1	1	1	1	1	0	1	0	7
**K. Gendzekhadze (2006)**	1	1	1	1	1	1	0	1	0	7
**Y. Li(2007)**	1	1	0	1	0	1	0	1	0	5
**TM. Lu(2007)**	1	1	0	1	1	1	0	1	0	6
**CJ. Li(2007)**	1	1	0	1	0	1	0	1	0	5
**P N. Nikolova (2008)**	1	1	1	1	1	1	0	1	0	7
**M A. Jiménez-Sousa (2012)**	1	1	1	1	1	1	0	1	0	7

### Quantitative synthesis

A total of 8 trials were included in the analysis of association between *TGFB1* polymorphisms and CAD. For *TGFB1* codon 10 T/C, no significant difference was found between CAD recipients and control subjects in all five models (TT vs. TC+CC: OR: 1.37, 95% CIs: 0.61, 3.06, *P* = 0.44; CC vs. TC+TT: OR: 1.36, 95% CIs: 0.77, 2.40, *P* = 0.29; TT vs. CC: OR: 1.15, 95% CIs: 0.36, 3.67, *P* = 0.82; TT vs. TC: OR: 1.24, 95% CIs: 0.52, 2.94, *P* = 0.63; T vs. C: OR: 1.29, 95% CIs: 0.78, 2.12, *P* = 0.32. Supplementary Figure 1). For *TGFB1* codon 25 G/C, there was no significant association between CAD recipients and control groups in dominant model, co-dominant model and allele model (GG vs. GC+CC: OR: 1.31, 95% CIs: 0.46, 3.74, *P* = 0.62; GG vs. GC: OR: 1.36, 95% CIs: 0.45, 4.12, *P* = 0.59; G vs. C: OR: 1.22, 95% CIs: 0.49, 3.02, *P* = 0.68. Supplementary Figure 2).

**Figure 2:**
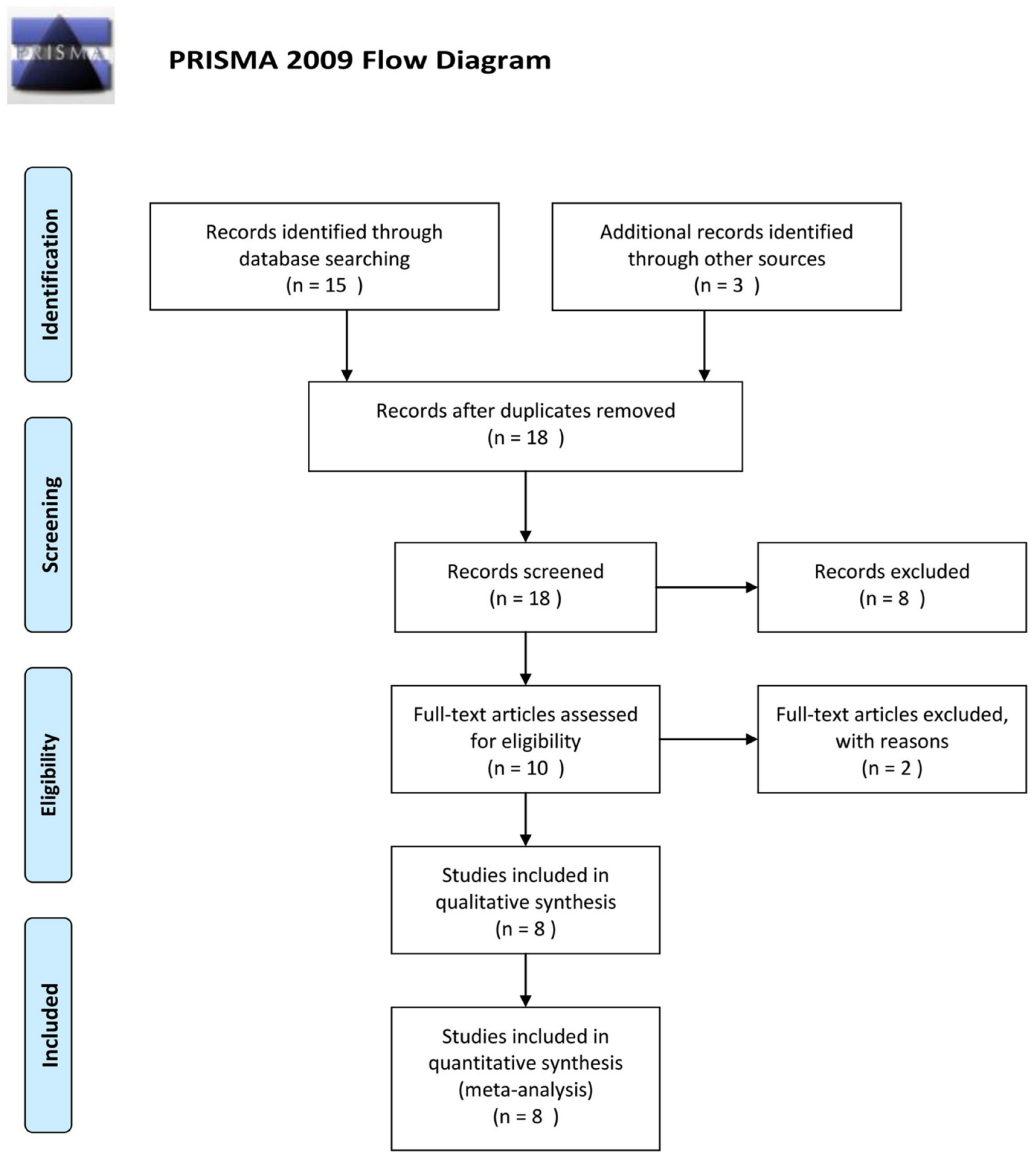
Meta-analysis of the association between the *TGFB1* haplotypes and CAD risk (HIGH vs. INTERMEDIATE + LOW)

Furthermore, we performed the meta-analysis to investigate the effect of *TGFB1* haplotype on the pathogenesis of CAD. According to the effects on TGF-β1 secretion, these haplotypes were categorized as “HIGH”, “INTERMEDIATE” and “LOW” (Supplementary Table 1) [16]. In summary, we observed statistically significant differences in *TGFB1* haplotypes between the CAD recipients and control subjects when comparing the “HIGH” with “INTERMEDIATE” and “LOW” (“HIGH” vs. “INTERMEDIATE” + “LOW”: OR: 3.56, 95% CIs: 2.20, 5.78, *P* < 0.001, Figure 2).

### Sensitivity analysis and publication bias

Sensitivity analysis was performed by sequential removal of individual studies to reflect the effect of individual data on the pooled ORs. No effect of any study was found on the pooled results in the above five models. The Begg’s funnel plot and Egger’s linear regression test was performed to determine publication bias. Results of funnel plot and regression test showed that there was no significant publication bias (Table 3).

**Table 3:** Begg’s funnel plot and Egger’s linear regression test for all included studies

genotypes	Egger’s test		Begg’s test	
	***t***	***P***	***z***	***P***
Codon 10				
TT vs. TC+CC	−1.15	0.33	0.49	0.62
CC vs. TC+TT	−0.23	0.85	0.52	0.60
TT vs. TC	−1.89	0.16	0.49	0.62
TT vs. CC	−0.08	0.95	−0.52	0.60
T vs. C	0.26	0.81	−0.49	0.62
Codon 25				
GG vs. GC+CC	0.93	0.52	0.52	0.60
GG vs. GC	0.40	0.76	0.52	0.60
G vs. C	1.00	0.50	0.52	0.60
Haplotype				
HIGH vs. INTERMEDIATE+LOW	2.13	0.28	−0.52	0.60

## DISCUSSION

The present meta-analysis included 8 studies with 1038 renal transplant recipients to assess the correlation between two *TGFB1* SNPs, codon 10 and 25, and CAD risk. In addition, this meta-analysis presented the first cumulative meta-analysis on this topic. In the cumulative meta-analysis, we found that “HIGH” haplotype, which contains codon 10, 25 T/T G/G and T/C G/G genotypes, was strongly associated with the development of CAD following kidney transplantation. However, no significant association of *TGFB1* SNPs in all five genetic models with CAD risk was observed.

TGF-β1 is a multifunctional cytokine that regulates the proliferation and differentiation of many cell types, and has been identified as an important promoter of fibrogenesis in various cells and tissues [26]. There is increasing evidence that TGF-β1 is strongly associated with the pathogenesis of interstitial fibrosis in kidney through a physical process, including EMT and EndMT [27]. The *TGFB1* SNPs +869T/C at codon 10 and +915G/C at codon 25 are important in the signal sequence and may influence the secretion of TGF-β1, contributing to the occurrence of CAD [13]. In our study, we found that the haplotype of *TGFB1* codon 10, 25 T/T G/G and T/C G/G genotypes, associated with higher production of TGF-β1, are more susceptible to CAD following kidney transplantation when compared with haplotypes of other genotypes. This is consistent with the results of studies conducted by P N. Nikolova [17].

The *TGFB1* codon 10 and codon 25 were associated with inter-individual variation in the level of TGF-β1 production. Dunning et al. (13) found that secretion of the proline form of TBG-β1 was 2.8 times that of the leucine form of TGF-β1 at codon 10 in transfected Hela cells, therefore the proline mutation at codon 10 could increase the amount of TGF-β1 protein secretion. Similarly, Cambien et al. [28] believed that the substitution of arginine with proline corresponded to a change from a large polar acid to a small apolar acid and concluded that this may affect the export of TGF-β1 protein. In our study, no significant association of *TGFB1* codon 10 or codon 25 with the CAD risk was found in all five models, suggesting that the single mutation in codon 10 and codon 25 were not responsible for the pathogenesis of CAD after kidney transplantation.

Notably, these results should be interpreted with caution. The case number of recipients with CAD included in our analysis is relatively small, which could lead to relatively high heterogeneity. Due to the limited number of studies in our analysis, we categorized CAN, confirmed by allograft renal biopsy, into the diagnosis of CAD, instead of subgroup analysis. Finally, there were at least 10 SNPs reported in the *TGFB1* gene, and except for codon 10 and codon 25, rare studies have focused on other SNPs, which may play a role in the pathogenesis of CAD.

In conclusion, our meta-analysis found the haplotype of *TGFB1* gene codon 10/25 T/T G/G and T/C G/G genotypes, associated with increased production of TGF-β1, was linked with CAD risk following kidney transplantation. Moreover, no significant difference was found between *TGFB1* codon 10 or codon 25 and the development of CAD. Further studies incorporating subjects with difference ethnic backgrounds combined with re-sequencing of the marked region and functional evaluations are warranted.

## SUPPLEMENTARY MATERIALS FIGURES AND TABLE


