# Supplementary material for: Association between TGFB1 genetic polymorphisms and chronic allograft dysfunction: a systematic review and meta-analysis
Source: Oncotarget. 2017 Jul 24;8(37):62463–9. doi: 10.18632/oncotarget.19516 (PMC5617520; doi:10.18632/oncotarget.19516)
Supplement: Supplementary file 1 [file oncotarget-08-62463-s001.pdf]

Association between *TGFB1* genetic polymorphisms and chronic allograft dysfunction: a systematic review and meta-analysis

SUPPLEMENTARY MATERIALS

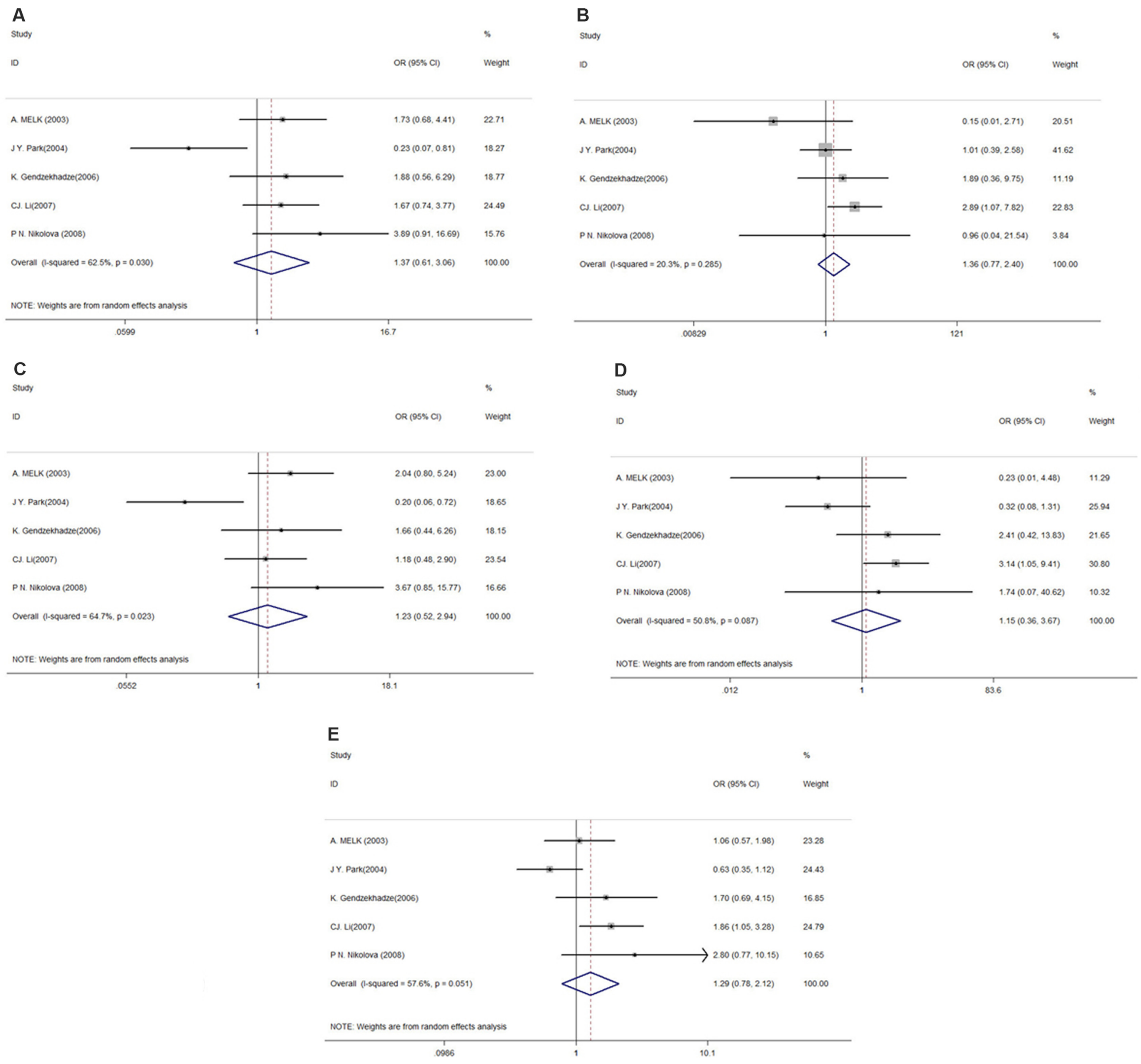

Supplementary Figure 1: Meta-analysis of the association between the *TGFB1* codon 10 and CAD risk in dominant model (**A**), recessive model (**B**), co-dominant model (**C**), co-recessive model (**D**) and allelic model (**E**).

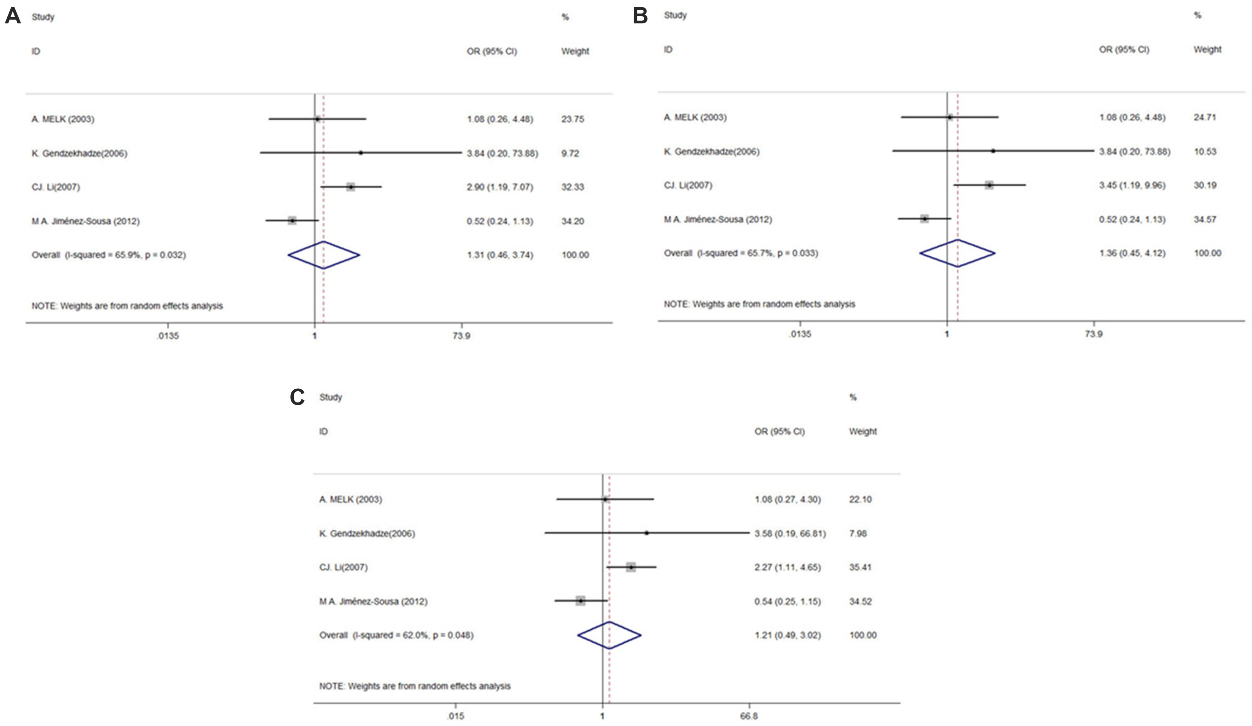

Supplementary Figure 2: Meta-analysis of the association between the *TGFB1* codon 25 and CAD risk in dominant model (**A**), co-dominant model (**B**) and allelic model (**C**).

Supplementary Table 1: Correspondence between TGFB1 cytokine gene polymorphism and level of TGF-β1 production

Cytokine polymorphism	Genotype	Level of production
**TGFB1 (Codon 10/25)**	T/T G/G, T/C G/G	HIGH
	T/C G/C, C/C G/G, T/T G/C	INTERMEDIATE
	C/C G/C, C/C C/C, T/T C/C,T/C C/C	LOW
